# The gateway reflex regulates tissue-specific autoimmune diseases

**DOI:** 10.1186/s41232-024-00325-6

**Published:** 2024-03-07

**Authors:** Yuki Tanaka, Izuru Ohki, Kaoru Murakami, Satoshi Ozawa, Yaze Wang, Masaaki Murakami

**Affiliations:** 1 Division of Molecular Psychoimmunology, Institute for Genetic Medicine, Graduate School of Medicine, Hokkaido University, Sapporo, Japan; 2 Quantumimmunology Team, Institute for Quantum Life Science, National Institute for Quantum and Radiological Science and Technology, Chiba, Japan; 3 Division of Molecular Neuroimmunology, Department of Homeostatic Regulation, National Institute for Physiological Sciences, National Institutes of Natural Sciences, Okazaki, Aichi Japan; 4 Institute for Vaccine Research and Development (HU-IVReD), Hokkaido University, Sapporo, Japan

**Keywords:** Tissue-specific autoimmune diseases, Multiple sclerosis, Gateway reflex, IL-6 amplifier

## Abstract

The dynamic interaction and movement of substances and cells between the central nervous system (CNS) and peripheral organs are meticulously controlled by a specialized vascular structure, the blood–brain barrier (BBB). Experimental and clinical research has shown that disruptions in the BBB are characteristic of various neuroinflammatory disorders, including multiple sclerosis. We have been elucidating a mechanism termed the “gateway reflex” that details the entry of immune cells, notably autoreactive T cells, into the CNS at the onset of such diseases. This process is initiated through local neural responses to a range of environmental stimuli, such as gravity, electricity, pain, stress, light, and joint inflammation. These stimuli specifically activate neural pathways to open gateways at targeted blood vessels for blood immune cell entry. The gateway reflex is pivotal in managing tissue-specific inflammatory diseases, and its improper activation is linked to disease progression. In this review, we present a comprehensive examination of the gateway reflex mechanism.

## Introduction

Endothelial cells within the brain and spinal cord possess apical junctional complexes, notably tight junctions, and, with astrocytes and other supporting cells, establish the blood–brain barrier (BBB). This barrier is integral to maintaining cerebral homeostasis and functionality of the central nervous system (CNS) through the regulation of molecular and cellular exchange between the CNS and peripheral regions. It also serves as a critical blockade against pathogen infiltration. Accordingly, BBB dysfunction has been implicated in a range of neurological disorders, including multiple sclerosis (MS).

Emerging hypotheses suggest that a spectrum of environmental stimuli, such as gravity and light, may influence cerebral stability and sometimes contribute to the pathogenesis of diseases through the activation of distinct neural pathways. Additionally, psychosocial interactions leading to psychological changes that encompass various forms of mental stress, anxiety, or even positive emotional states are recognized as environmental stimuli. A well-characterized mechanism in stress responses involves the secretion of corticosteroid hormones and is mediated by the hypothalamus–pituitary–adrenal (HPA) axis, which systematically modulates immune responses [1, 2]. Beyond these systemic effects, our 2012 findings revealed the localized regulation of inflammatory states through specific neural activations, an interaction we termed the “gateway reflex” [3]. In this context, a gravitational response induced by sensory neural stimulations in the soleus muscles prompts chemokine production in the dorsal blood vessels of the fifth lumbar (L5) region, but not in other spinal segments, via specific sympathetic nerve activation. In the model of experimental autoimmune encephalomyelitis (EAE), a mouse analog for MS, such an upregulation of chemokines, facilitates the incursion of CNS-reactive CD4 + T cells (deemed pathogenic CD4 + T cells) through the L5 dorsal vessels [3]. This intersection of the neural and immune systems prompted the hypothesis that specific neural pathways exert a localized influence on immune reactions and organ function. While our initial findings highlighted pro-inflammatory outcomes from local sensory-sympathetic interactions, other groups, including Dr. Kevin Tracey’s team, have documented anti-inflammatory responses elicited through vagal nerve stimulations, referred to as the inflammatory reflex [4–7]. This review article encapsulates such distinct neuro-immune interactions, which are instrumental in regulating tissue-specific inflammatory diseases.

### CNS inflammation and BBB

The BBB is constituted of an intricate network of cell–cell interactions primarily among endothelial cells, pericytes, and astrocytes. A key component of this network is the tight junction, which is pivotal in maintaining the separation between blood and cerebrospinal fluid and is facilitated by molecules like claudins and occludins [8]. Despite its intricate design, the BBB is not impervious and allows the infiltration of immune cells into the CNS, which can lead to autoimmune conditions such as MS [9, 10]. The infiltration has also been linked to neurodegenerative diseases like Alzheimer’s disease [11, 12]. Inflammatory processes, which are marked by cytokines, such as IL-1β, IL-17A, IFNα, and TNFα, compromise the integrity of the BBB, thereby enhancing its permeability [13–15]. MS, which is characterized by demyelination affecting motor, sensory, autonomic, and cognitive functions, emerges as a result of both genetic and environmental factors [9]. This conclusion is evidenced by genome-wide association studies and analyses of single-nucleotide polymorphisms, which have identified specific alleles related to major histocompatibility complex class II and genes associated with CD4 + T-cell activation as factors in MS development [9, 16]. Animal models have further substantiated these findings. Therefore, targeting the activation and migration of pathogenic autoreactive CD4 + T cells into the CNS is a promising therapeutic strategy for MS. Existing treatments, such as fingolimod and natalizumab, have shown efficacy in managing MS by targeting CD4 + T cell migration [17–19]. However, these treatments are not without side effects, including the risk of progressive multifocal leukoencephalopathy due to systemic immune suppression.

## Gravity gateway reflex

**Fig. 1:**
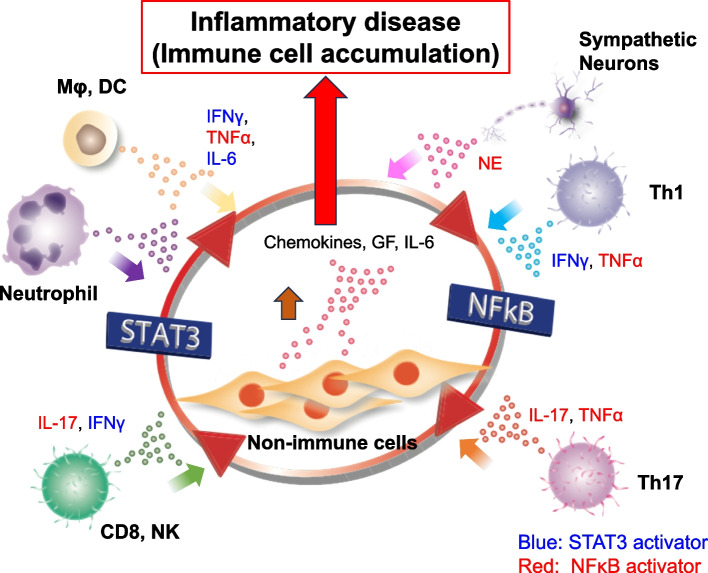
The IL-6 amplifier. The IL-6 amplifier describes the simultaneous activation of NF-kB and STAT3 in nonimmune cells, leading to a significant production of chemokines and cytokines. Consequently, NF-kB activity is heightened, and the pathogenesis of various inflammatory diseases emerges due to an excessive inflammatory response. MΦ, macrophages; DC, dendritic cells; NK, natural killer cells; CD8, CD8 + T cells; Th17, Th17 cells. Regarding the gateway reflex, the IL-6 amplifier is activated in vascular endothelial cells near activated local nerves following the section of norepinephrine (NE) from sympathetic nerves

**Fig. 2:**
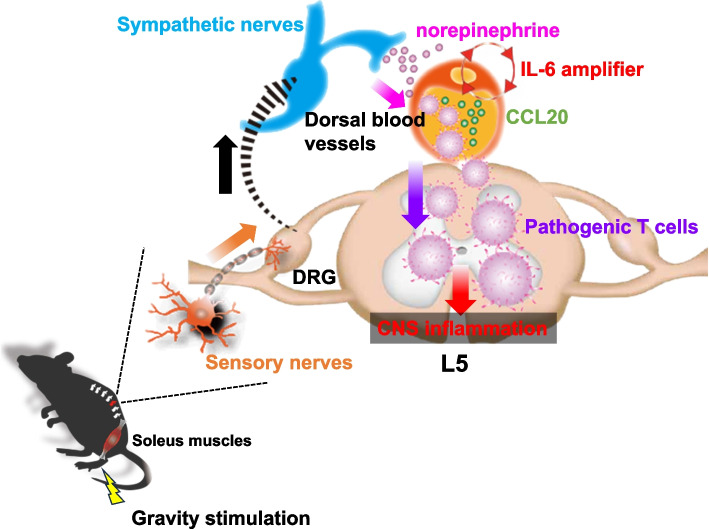
The gravity gateway reflex. Gravity stimulates sensory nerves in the soleus muscles to activate sympathetic nerves connected to the L5 dorsal vessels. Norepinephrine released by these sympathetic nerves targets L5-specific vessels and activates the IL-6 amplifier, culminating in the accumulation of pathogenic CD4 + T cells in the L5 spinal cord, an event primarily mediated by CCL20. Sensory neurons and sympathetic neurons are thought to be connected in the L5 DRG

The clinical manifestations of MS depend on the locations of the inflammatory lesions, which are often initiated by myelin-specific CD4 + T cells. The variability in lesion sites across patients points to an undefined mechanism of CNS invasion by these cells. Our research utilized an adoptive transfer model of EAE to investigate the initial entry points. An immunohistochemical analysis revealed a predominant accumulation of pathogenic CD4 + T cells in the dorsal vessels of the L5 spinal cord that was distinguished by elevated levels of chemokines, such as CCL20, which attract Th17 cells, a subtype of CD4 + T cells implicated in the EAE pathogenesis. This enhanced chemokine production is stimulated by the concurrent activation of the NF-κB and STAT3 pathways through a mechanism we call the IL-6 amplifier [20–27] (Fig. 1), which is implicated in various inflammatory diseases. Treatments targeting CCL20 or employing CCR6-deficient CD4 + T cells have been shown to suppress the accumulation of pathogenic CD4 + T cells in the L5 spinal cord. Interestingly, even without EAE induction, L5 dorsal vessels exhibited higher chemokine levels compared to those in the L1 cord, suggesting an inherent property of these vessels in both normal and diseased states. Neurons in the L5 dorsal root ganglion linked to the soleus muscles, which are the primary antigravity muscles, are thought to be activated by gravity. This fact led us to hypothesize that gravitational stimuli on these muscles trigger chemokine production in L5 dorsal vessels, thus forming an initial gateway for pathogenic CD4 + T cells in the bloodstream. *Tests employing NASA’s ground simulations *[28–30], where mice are tail-suspended to relieve the hind legs from gravity, showed a reduced chemokine expression in the L5 dorsal vessels and diminished accumulation of pathogenic CD4 + T cells. Conversely, electrical stimulation of the soleus muscles in these suspended mice restored the chemokine expression and CD4 + T-cell accumulation at L5. These findings strongly suggest that regional sensory neural activation by gravity plays a significant role in mediating local inflammation at L5 dorsal vessels, especially in the presence of pathogenic CD4 + T cells, constituting a novel neuro-immune interaction dubbed the “gateway reflex.” Further investigations revealed heightened c-Fos levels in L5 sympathetic *dorsal root ganglions (DRG)* compared to those in L1, indicating the involvement of sympathetic nerves. Pharmacological interventions that inhibit sympathetic nerve activity have been shown to suppress EAE development by reducing chemokine production and the accumulation of pathogenic CD4 + T cells at the L5 dorsal vessels. *However, it is unknown how gravity regulates the connection of sensory neurons *via* the soleus muscles to sympathetic neurons in L5 sympathetic ganglion. Although sympathetic axons are present in normal DRG, their number increases after injury to sensory neurons, suggesting that this connection occurs in L5 DRG *[31]. Hence, the gravity gateway reflex encompasses local sensory-sympathetic communications essential for gateway formation at the L5 dorsal vessels [3] (Fig. 2).

### Electric gateway reflex

The gateway reflex is not confined to the soleus-L5 axis. It has been observed that stimulating regional neurons connected to various muscles can induce the formation of gateways in dorsal vessels along different spinal cord levels. For instance, electrical stimulation of the quadriceps leads to an upregulation of chemokines, including CCL20, in the dorsal vessels at the L3 level. Similarly, electrical stimulations of muscles in the upper body, such as those in the forefoot, result in increased chemokine expression in the dorsal vessels of the cervical to thoracic regions of the spinal cord. This phenomenon, where the immune cell gateway in the BBB is artificially controlled, has been termed the electric gateway reflex. These findings indicate the potential for artificial manipulation of the gateway reflex, opening avenues for therapeutic applications, including novel drug delivery systems targeting inflammatory diseases within the CNS.

### Pain gateway reflex

Our focus then shifted to pain sensation, which is recognized as a continuous sensory stimulation and a prevalent, detrimental symptom adversely affecting the quality of life in various pathologies. A notable correlation exists between the severity of MS and the incidence of pain, with sensitization to pain sensation often reported during the progression of EAE with expansion of the *inflamed area* [32, 33]. In the adoptive transfer model of EAE, recipient mice exhibit paralysis approximately 10 days after the transfer of pathogenic CD4 + T cells, followed by a phase of symptom recovery [34]. To explore the influence of pain on EAE symptoms, pain *sensation* was induced in mice through ligation of the middle branch of the trigeminal nerves, which are composed solely of sensory nerves. Mice with pathogenic CD4 + T cells and nerve ligation exhibited significantly worsened EAE symptoms, while the administration of pain medication attenuated the development of EAE [35]. Thus, pain serves not only as an indicator of the disease status but also plays a contributory role in the progression of EAE. Given that a significant proportion of MS patients experience symptom relapse, often accompanied by heightened pain in cases with more severe disease, the effect of pain induction was also examined during the remission phase of EAE. In line with this concept, mice that had recovered from EAE symptoms exhibited a relapse of paralysis following a trigeminal nerve ligation or the injection of pain-inducing substances like substance P and capsaicin. Initially, during the first episode of EAE, the dorsal vessels of the L5 spinal cord serve as the primary gateway for immune cells. To identify the immune cell gateway during the pain-induced relapse, an immunohistochemical analysis was conducted on EAE mice in the remission phase. This analysis revealed a significant presence of periphery-derived activated monocytes with high MHC class II expression around the meningeal region of the L5 cord. Interestingly, following the pain induction, these monocytes with high MHC class II accumulated prominently at the ventral vessels of the L5 cord. Additionally, norepinephrine (NE) signaling was observed in this area, with MHC class II high CD11b + monocytes secreting CX3CL1 in response to NE stimulation, at least in vitro. This observation suggests the presence of an autocrine or paracrine loop for the accumulation of these monocytes. Subsequently, pathogenic CD4 + T cells from the bloodstream invaded the spinal cord parenchyma from the L5 ventral vessels, indicating these vessels are the gateway during pain-induced relapse in the EAE model. *We also found that pain sensation mainly activates Nav1.8* + *sensory neurons, which is followed by activation the anterior cingulate cortex (ACC), a pain center where the sensory pathway transmits to the sympathetic pathway toward ventral vessels of the spinal cord including the L5 level. Indeed, blocking ACC activation suppressed the EAE relapse, while ACC activation even without pain induced periphery-derived activated monocytes with high MHC class II accumulation and EAE relapse*. The suppression of pain-induced EAE relapse can be achieved through genetic deficiency or pharmacological inhibition of the pain sensory pathway, suggesting a role for the sensory-sympathetic axis, similar to the gravity gateway reflex [35].

### Stress gateway reflex

The adage “illness starts in the mind” reflects the common experience where chronic stress often leads to gastrointestinal disorders via the brain-gut axis, though the underlying molecular mechanisms remain elusive. Given the association of stress with neural activation in brain regions, such as the paraventricular nucleus (PVN), dorsomedial nucleus of the hypothalamus (DMH), and dorsal motor nucleus of the vagus nerve (DMX), we proposed that chronic stress initiates a specific brain-based gateway reflex. In our studies, EAE under chronic stress conditions, like sleep disturbance, resulted in severe gastrointestinal dysfunction and heightened mortality [36]. Normally, pathogenic CD4 + T cells accumulate in the dorsal vessels of the L5 cord due to the gravity gateway reflex. However, under stress, these cells infiltrated specific vessels in the brain’s third ventricle boundary area, thalamus, and dentate gyrus, leading to localized brain inflammation, but not in the L5 cord. This observation indicates that chronic stress can redirect the immune cell gateway to the brain. In EAE mice under chronic stress, chemokine levels increased significantly in the gateway vessels but decreased in the L5 dorsal vessels. This observation suggests the enhancement of a unique neural pathway involving brain nuclei like the PVN, DMH, and DMX. Neural tracing revealed connections, particularly TH + noradrenergic connections, from the PVN to the gateway vessels and from the vessels to the DMH. The activation of the PVN, a key stress signal integrator, likely affects the gateway vessels via the newly identified neural circuit. In stressed mice, chemokines like CCL5 were elevated in the gateway vessels, attracting pathogenic CD4 + T cells and leading to micro-inflammation. This inflammation involved substances, such as ATP, serving as both an inflammatory mediator and neurotransmitter. Using an ATP receptor antagonist at the gateway vessels markedly reduced neural activation in the DMH region and significantly lowered mortality in EAE mice with stress. Moreover, mitigating brain micro-inflammation through cytokine neutralization or neural pathway disruption reduced gastrointestinal failure and improved survival rates. Investigating whether micro-inflammation alone suffices for stress-induced EAE phenotypes, we found that the intracerebroventricular injection of cytokines or ATP into these vessels induced severe gastrointestinal failure in stressed mice. Furthermore, reducing gastrointestinal inflammation with a proton-pump inhibitor also improved survival, suggesting that such inflammation upstream leads to sudden death. These findings demonstrate that brain micro-inflammation in specific vessels can activate resting neural pathways through ATP production, with ATP-induced neural activation in the DMH region exacerbating stress responses and causing severe gastrointestinal damage via the DMX and vagal nerve activation. Further, they establish a direct link between brain micro-inflammation and gastrointestinal homeostasis under stress, leading us to define the phenomenon known as the stress gateway reflex [36].

**Fig. 3:**
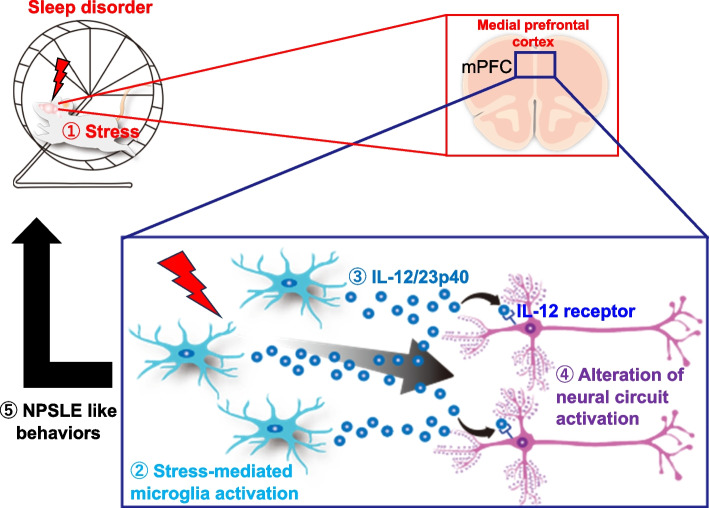
Stress-mediated neuropsychiatric systemic lupus erythematosus (NPSLE) mouse model. A cartoon of the mouse model. (1) Stress is induced by sleep deprivation (SD). (2) Neurons in the mPFC are activated, leading to the activation of microglia. (3) IL-12/23p40 is produced from the microglia. (4) IL-12/23p40 induces the activation of neurons in the mPFC to abnormally activate neural circuits, leading to (5) neuropsychiatric phenotypes. mPFC, medial prefrontal cortex

To explore another stress-induced disease model, we focused on systemic lupus erythematosus (SLE), a chronic autoimmune disease affecting multiple organs. Approximately half of SLE patients exhibit neurological and psychiatric symptoms, classified as neuropsychiatric SLE (NPSLE) [37]. NPSLE is further categorized into focal neurological syndrome-type NPSLE (fNPSLE) and diffuse neurological-psychiatric-cognitive syndrome-type NPSLE (dNPSLE), with dNPSLE being diagnosed in a majority of NPSLE patients and characterized by a range of clinical manifestations [38]. It is theorized that dysregulated neural circuits due to autoinflammatory conditions in the CNS play a role, but a specific mouse model for NPSLE is lacking. In contrast, several mouse models for human SLE exist. *We employed MRL/MpJ with FAS deficiency (MRL-lpr) mice as a representative SLE model. Although MRL-lpr mice spontaneously develop autoimmune infiltration and systemic inflammation normally *[39, 40]*, some CNS symptoms, such as the NPSLE-like acute confusional state, cognitive dysfunction, anxiety, and depression, are observed. We established an NPSLE mouse model by imposing a relatively mild stress, sleep disturbance, on MRL-lpr mice; MRL/MpJ mice without FAS deficiency showed no disease phenotype with sleep disturbance* [41] (Fig. 3).

*Using a behavioral test, elevated plus maze test, and open field test to exam risk-taking agitation-like behaviors, we confirmed chronic stress leads MRL-lpr mice to show the acute confusional state, such as psychomotor overactivity and vigilance, seen with patients *[42].

Under chronic stress, such as sleep disturbance, these mice exhibit behaviors indicative of stress, with an increased expression of the pro-inflammatory cytokine gene IL-12-/23 p40 in the medial prefrontal cortex (mPFC), a region crucial for managing behavioral and psychological stress. The increased expression of IL-12/23 p40 in the CSF and mPFC atrophy in dNPSLE patients suggests a neural and microglial hyperactivation similar to that in stressed MRL-lpr mice [41]. Treatment with IL-12 receptor-neutralizing antibodies or tyrosine kinase 2 inhibitors ameliorated abnormal behaviors and dendritic spine hyperplasia, suggesting a potential therapeutic target in dNPSLE. Our findings thus draw a parallel between dNPSLE and the stress-exposed MRL-lpr model, both immunologically and clinically, and highlight IL-12/IL23 p40 production by microglia in the mPFC as a potential therapeutic target [41].

## Light gateway reflex

The light gateway reflex describes a neural circuit-based inhibitory mechanism for inflammatory diseases. Uveitis, an autoimmune condition characterized by inflammation and tissue damage in the retina and choroid mediated by pathogenic CD4 + T cells, can lead to severe visual impairment and blindness. The experimental model for this disease, experimental autoimmune uveoretinitis (EAU), is established in mice through immunization with intercellular retinoid-binding protein (IRBP) or the introduction of pathogenic CD4 + T cells recognizing IRBP [43–46]. These cells cross the blood-retina barrier, instigating inflammatory pathology within the retina. In our studies, housing EAU mice in photopic light conditions during the daytime markedly reduced the inflammatory phenotype. This effect was attributed to the suppression of α1A-adrenergic receptor expression in cells of the retinal blood vessel, or the gateway vessel, a consequence of a transient rise in NE levels in the eyes. This increase in NE rendered these vessel cells less responsive to NE, thereby diminishing NF-kB-mediated inflammation and cytokine production and preventing activation of the IL-6 amplifier. Our findings thus highlight the protective role of photopic illumination against the invasion of pathogenic CD4 + T cells into retinal tissues, thereby averting neuroinflammation and tissue damage [47].

### Remote inflammation gateway reflex

**Fig. 4:**
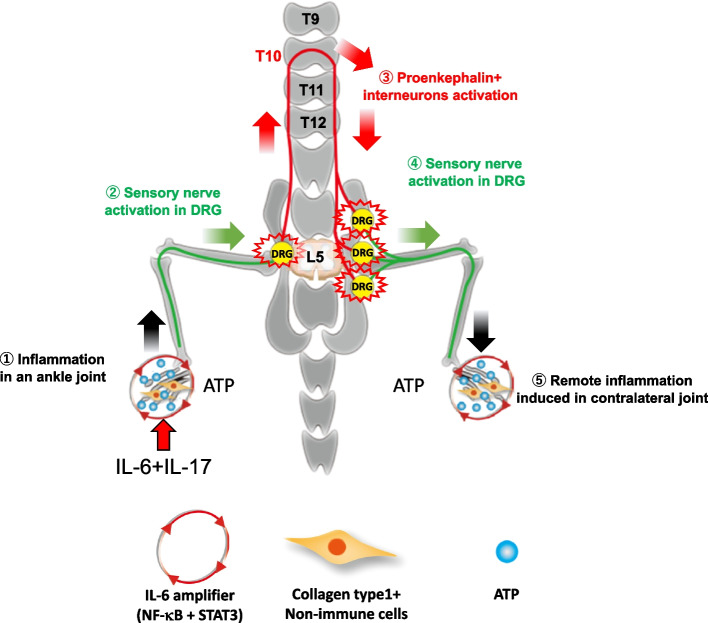
Stages of the remote inflammation gateway reflex. (1) ATP is highly secreted from nonimmune cells with activation of the IL-6 amplifier in an ankle joint. (2) ATP stimulates Nav1.8 + sensory neurons in the L5 DRG. (3) L5 sensory neurons signaling to proenkephalin-positive interneurons in the spinal cord between L5 and T10. (4) Sensory neurons in the contralateral L4-6 DRG are activated. (5) The activated sensory neurons secrete ATP antidromically in the contralateral ankle joint, leading to activation of the IL-6 amplifier, which is critical for the development of remote inflammation

Joint inflammation in rheumatoid arthritis patients often manifests bilaterally along affected joints [48, 49]. This bilateral spread of inflammation has been replicated in various animal models of rheumatoid arthritis. Previous studies have indicated the significance of neural pathways in the bilateral propagation of inflammation, but detailed insights into the specific neural pathways and molecular mechanisms are unestablished. Our recent research uncovered a molecular mechanism that features ATP-dependent neural activation to connect both ankle joints in two animal models. This activation is essential for the symmetric propagation of inflammation and represents a mechanism we termed the remote inflammation gateway reflex [50] (Fig. 4). In F759 mice, a model for spontaneous rheumatoid arthritis-like disease, arthritis is rapidly induced by IL-6 and IL-17A injections into the ankle joints. The remote inflammation gateway reflex is characterized by neural communication between sensory and interneurons mediated by ATP, which functions both as a neurotransmitter and an IL-6 amplifier enhancer. To investigate the transmission of the inflammation-induced neural activation through neural circuits to peripheral sites, we employed F759 mice and a type II collagen-induced arthritis (CIA) model, priming both models with arthritic inflammation and neural activation at the ankle joints. ATP, which is produced by nonimmune cells during inflammation, activated sensory pathways. The resulting neural circuits, comprising Nav1.8 + sensory neurons and proenkephalin + interneurons, extended to the contralateral ankle joints. ATP released antidromically from sensory axons triggered inflammation through the IL-6 amplifier. The application of an ATP receptor antagonist (A438079) or surgical disruption of the sensory and interneuron networks substantially mitigated symmetrical joint inflammation in both models [50]. *Although symmetric remote inflammation is one of the definitive criteria of rheumatoid arthritis, another feature is chronic pain, suggesting a relationship between the remote inflammation gateway reflex and the pain gateway reflex. Therefore, we suppressed ACC activation and found the remote inflammation gateway reflex was normally induced. This observation argues little connection between the two reflexes*.

## Conclusion

This review presents a novel paradigm in the field of tissue-specific inflammatory diseases, highlighting a unique neuro-immune interaction. We have identified a mechanism that facilitates the entry of autoreactive T cells into the BBB and triggers inflammatory processes. This mechanism involves chemokine expression at specific vascular sites initiated by the activation of a specialized neural circuit termed the “gateway reflex.” While traditional approaches in neuro-immune cross talk have focused on systemic immunosuppression through glucocorticoid regulation, because they often involve steroid therapies, they raise concerns about potential side effects. In contrast, the gateway reflex, rooted in neural circuitry, offers a more localized approach to immune control, potentially reducing the likelihood of adverse reactions and avoiding systemic immunodeficiency.

The gateway reflex demonstrates that environmental factors, such as gravity [3], pain [35], stress [36, 41], light [47], and local inflammation [50], can modulate the activation sites of local nerves. This modulation, in turn, influences the innervation of specific blood vessel sites including the BBB. *Genetic factors particularly in patients also should influence the gateway reflex considering individual variability in phenotypes. Indeed, disease-associated genes are involved in the activation of the IL-6 amplifier, and risk alleles at sQTL or eQTL respectively increase the alternative splicing and change the RNA level of disease-associated genes *[24, 27, 51].

Finally, with a focus on the tissue specificity of diseases, it is therefore crucial *to understanding the gateway reflex*. The influence of neurotransmitters from the nervous system on both immune [52–55] and nonimmune cells [3, 35] throughout the body underscores the broad implications of the gateway reflex. *Many biological agents, including antibody drugs, have been developed and used for the treatment of autoimmune diseases and other inflammatory diseases. However, refractory cases still exist, and there is no definitive treatment that overcomes all cases. Because the gateway reflex is a tissue-specific mechanism that regulates local neuronal activation. It may be possible to open and close the gates of the gateway reflex in specific neuronal circuits using “neuromodulation,” a technology for artificial neural stimulation, thereby controlling the disease. Thus, neuromodulation of the gateway reflex circuits holds significant promise for the development of therapeutic strategies targeting a range of inflammatory diseases, offering a new avenue for treatments that leverage the intricate interplay between the nervous and immune systems*.

## Data Availability

Not applicable.

## Abbreviations

CNS: Central nervous system
BBB: Blood-brain barrier
MS: Multiple sclerosis
HPA: Hypothalamus-pituitary-adrenal grand
L5: Fifth lumbar
EAE: Experimental autoimmune encephalomyelitis
MHC: Major histocompatibility complex
Th17 cells: IL-17-secreting type-17 CD4 + T cells
DRG: Dorsal root ganglion
NASA: National Aeronautics and Space Administration
PVN: Paraventricular nucleus
DMH: Dorsomedial nucleus of hypothalamus
DMX: Dorsal motor nucleus of the vagus nerve
SLE: Systemic lupus erythematosus
NPSLE: Neuropsychological SLE
fNPSLE: Focal neurological syndrome-type NPSLE
dNPSLE: Diffuse neurological-psychiatric-cognitive syndrome-type NPSLE
MRL-lpr: MRL/MpJ with FAS deficiency
mPFC: Medial prefrontal cortex
EAU: Experimental autoimmune uveoretinitis
IRBP: Intercellular retinoid-binding protein
CIA: Type II collagen-induced arthritis
NE: Norepinephrine
